# Tunable Switching Behavior of GO-Based Memristors Using Thermal Reduction

**DOI:** 10.3390/nano12111812

**Published:** 2022-05-25

**Authors:** Muayad Abujabal, Heba Abunahla, Baker Mohammad, Anas Alazzam

**Affiliations:** 1 System on Chip Lab, Department of Mechanical Engineering, Khalifa University, Abu Dhabi P.O. Box 127788, United Arab Emirates; 100057733@ku.ac.ae; 2 System on Chip Lab, Department of Electrical Engineering and Computer Science, Khalifa University, Abu Dhabi P.O. Box 127788, United Arab Emirates; heba.abunahla@ku.ac.ae (H.A.); baker.mohammad@ku.ac.ae (B.M.)

**Keywords:** graphene oxide, reduction, thermal, memristor, switching, analog, oxidation

## Abstract

This work reports on the fabrication of a novel planar reduced graphene oxide (rGO) memristor (MR) device. For the first time in the literature, the MR tunable resistive switching behavior is controlled by the GO reduction time at a constant temperature. The device is fabricated using standard microfabrication techniques on a flexible cyclic olefin copolymer substrate (COC). Thermal reduction of the GO layer at low temperatures (100 °C) avoids the drawbacks of chemical reduction methods such as toxicity and electrode metal damage during fabrication, while allowing for fine-tuning of the MR’s switching behavior. The device has analog switching characteristics, with a range of different resistance states. By taking advantage of the slow nature of GO thermal annealing, the switching properties of the rGO memristors can be precisely controlled by adjusting the reduction period. At short annealing times (i.e., T < 20 h), the devices switch from high to low resistance states, while at longer annealing times the switching behavior is reversed, with the device switching from low to high resistance states (LRS to HRS). Resistive switching occurs as a result of the diffusion and removal of the oxygen functional groups in the GO film caused by Joule heating induced by the electric current. Complete electrical characterization tests are presented along with wettability and X-ray diffraction (XRD) tests. This work opens a new vision for realizing rGO-based MR devices with tunable switching properties, broadening the application horizon of the device.

## 1. Introduction

The memristor device (MR) is a two-terminal electrical component that was first proposed by Leon Chua in 1971 as the fourth fundamental circuit element that connects electric charge and magnetic flux [1]. MR has a closed hysteresis *I-V* characteristic that is pinched at zero and can switch between two resistance states. The low-resistance state is referred to as the “ON state” while the high-resistance state is referred to as the “OFF state”. HP labs fabricated the first MR in 2008, using TiO_2_ as the active switching layer [2]. After this, the interest in MR devices has dramatically increased as their unique features make them a great candidate for many emerging applications such as memory, computing, security, and sensing [3,4,5,6].

The sensing applications of MR have gained momentum due to the great ability of the device to change its fingerprint *I-V* characteristics according to the surrounding environments and the interactions taking place in the device’s active area. Tzouvadaki et al. [7,8] developed a bio-functionalized silicon nanowire MR for cancer detection by attaching anti-PSA antibodies; the sensor functioned by exhibiting a voltage gap, or a non-zero current at zero volts. Additionally, the silicon nanowire was used to develop a sensor for drug monitoring in a manner similar to that used in cancer detection [9]. The silicon nanowire is analogous to enzymatic sensors in which biomolecules are attached to the device [10]. MR devices have been used as non-enzymatic sensors, and a device based on TiO_2_ fabricated as a crossbar was used to detect glucose at concentrations comparable to those found in human blood samples by tracking changes in the ON/OFF ratio [11]. These applications demonstrate MR’s versatility and its suitability to be deployed in biosensing applications. Device structure is a key factor for deploying MR devices in sensing applications as it should facilitate the interaction between the device’s active area and the surrounding environment or the applied molecules. Thus, planar MR devices are considered a great asset for sensing applications.

In recent years, graphene and graphene-related compounds have garnered considerable attention [12]. Concurrently, graphene-based resistive switching devices have been developed [13]. In these devices, graphene and graphene oxide (GO) are used to fabricate the electrodes as well as the active resistive switching material [13,14,15]. For instance, GO has been used as an active layer in the development of artificial neural networks (ANNs) that mimic the behavior of neurons and the way the human brain retains information [3]. Moreover, reduced GO (rGO)-based memristors have also been used in the development of sensors [5]. GO is a two-dimensional substance formed from graphene that is decorated with oxygen functional groups [16]. The low cost and ease of manufacturing make GO especially attractive. GO-based memristor devices can be produced using a variety of standard fabrication procedures. GO can be used in its natural state [17] or it can be changed by chemical, electrochemical, thermal, or photothermal processes [18].

To the best of our knowledge, this is the first study to examine thermal reduction for the fabrication of planar rGO-based MR devices on a flexible polymer substrate. It details the development of a low-cost planar MR device that consists of Au-rGO-Au. On top of a cyclic olefin copolymer (COC) substrate, the electrodes and rGO layer are stacked in a planar metal/GO/metal configuration. The device is fabricated using standard microfabrication techniques, with the GO layer being thermally reduced at a constant temperature. Planar devices benefit from their simplicity of fabrication, which permits mass production and incorporation into low-cost disposable electronics. Due to its adaptability in applications such as flexible sensors and wearable devices, flexible electronics are gaining appeal [19]. Additionally, the planar structure has a large surface area, which provides more space for the electrochemical reactions [20]. In this work, the MR switching behavior of the device is controlled at low annealing temperatures (100 °C). This results in increased biocompatibility and safety during fabrication when compared to other reduction methods of GO. Thermal annealing at a low temperature has critical advantages over chemical reduction, which is ineffective when acid-sensitive metals are used. Furthermore, unlike previous approaches, it does not necessitate direct access to the GO film. Additionally, it requires less equipment than photothermal and chemical techniques. By carefully tweaking the reduction process, different electrical properties can be achieved for a variety of applications, ranging from computation to sensing. The obtained devices displayed unipolar analog switching characteristics with controllable switching behavior due to the degree of reduction of the GO film between the two gold electrodes. By varying the degree of reduction of the GO layer, two distinct device behaviors were achieved. Adjusting the duration of heating is sufficient for regulating the reduction process. The resulting resistive switching behavior has a great potential for a variety of applications in different fields; memristive sensors exploit the resistive switching ability to detect various quantities and substances. The thermal reduction approach of graphene oxide facilitates the construction of biocompatible sensors to detect biomolecules in enzymatic or non-enzymatic approaches. Additionally, the memristor exhibits an analog switching behavior when GO is highly reduced, and the multiple resistance states make it a prime candidate to build multi-bit memory cells for high density memory. Additionally, it can support in-memory computing and neuromorphic computing, especially for artificial neural networks (ANN).

## 2. Materials and Methods

### 2.1. Device Fabrication

**Figure 1:**
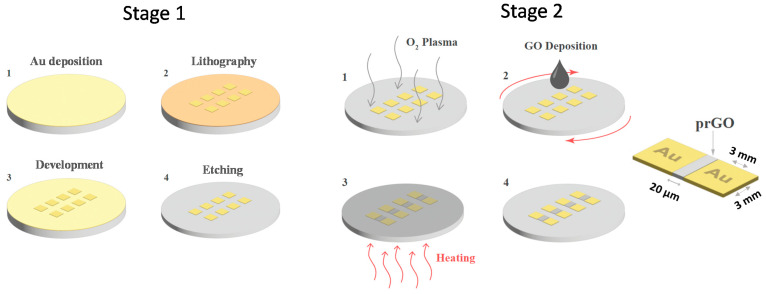
Memristor device fabrication steps. Stage 1 includes the patterning of the gold electrodes, while Stage 2 involves the deposition of the GO layers between the planar electrodes.

As shown in Figure 1, the planar MR devices reported in this work are fabricated using standard microfabrication techniques. The lithography photomask is fabricated using a direct photolithography system (Dilase Kloe 650, Kloe-france, Saint-Mathieu-de-Tréviers, France). The electrodes are designed to be square in shape, 3 mm long, and separated by a 20 μm gap, as illustrated in Figure 1. The first stage of the device fabrication includes the deposition of a thin layer of gold using a sputter (Q300TT from Quorum, Laughton, East Sussex, United Kingdom) on a clean cyclic olefin copolymer substrate (COC) (TOPAS Advanced Polymers 5013 from microfluidic ChipShop, Jena, Germany) (Step 1). Following that, a layer of photoresist (PR1813 from micro resist technology GmbH, Berlin, Germany) is deposited using a spin coater (WS650Hzb-23NPP UD-3 from Laurell Technologies Corporation, North Wales, PA, United State) and then baked for five minutes at 70 °C. The wafer is then subjected to ultraviolet light via the photomask (step 2). The photoresist patterned layer is then developed using a suitable developer, washed in DI water, and then the gold layer is etched with gold etchant (TechniEtch ACI2 potassium iodide/iodine etchant from Microchemicals GmbH, Ulm, Germany) (steps 3 and 4). Following acetone removal of the photoresist layer, the wafer is thoroughly washed with DI water. After patterning the electrodes, the GO (Graphenea, San Sebastián, Spain) layers are deposited (Stage 2). The deposition of GO begins with a five-minute oxygen plasma treatment (PDC-002 from Harrick Plasma, Ithaca, NY, United States) of the COC substrate to improve the adherence of GO flakes and the surface’s wettability [21] (step 1). Then, spin coating is used to deposit a thin layer of GO on the substrate (step 2). The wafer with GO layers is then baked for two minutes at 70 °C on a hot plate. The GO deposition (on top of the substrate) and baking steps are carried out three times (steps 3 and 4). It is important to note that the patterned electrodes are protected with scotch tape during GO deposition. In this work, the reduction of GO is achieved by heating the device at 100 °C in an air environment. As described in Section 3, the heating time is adjusted and its effect on the memristor device switching behavior is studied and analyzed.

### 2.2. Device Characterization

Microphotographs of the fabricated memristor devices are captured using a field emission scanning electron microscope (FESEM) (JEOL, JSM-7610F, Tokyo, Japan). In addition, detailed material characterizations utilizing an X-ray diffractometer (XRD) (Bruker, D2 Phaser, Billerica, MA, USA), Raman spectroscopy and atomic force microscopy (AFM) are performed on the devices. Detailed electrical characterization is performed using Keithley 4200-SCS parameter analyzer (from Tektronix, Beaverton, OR, USA). One electrode is connected to the biasing terminal, while the second electrode is kept grounded. The voltage sweeping mode is used to measure the current-voltage (*I-V*) characteristics of the fabricated devices, with a voltage step of 0.05 V and range up to 15 V. Additionally, the pulse mode is utilized to study the effect of applying constant voltage over time on the device switching behavior.

## 3. Results and Discussion

### 3.1. Material Characterization

#### 3.1.1. Wettability and Contact Angle

**Figure 2:**
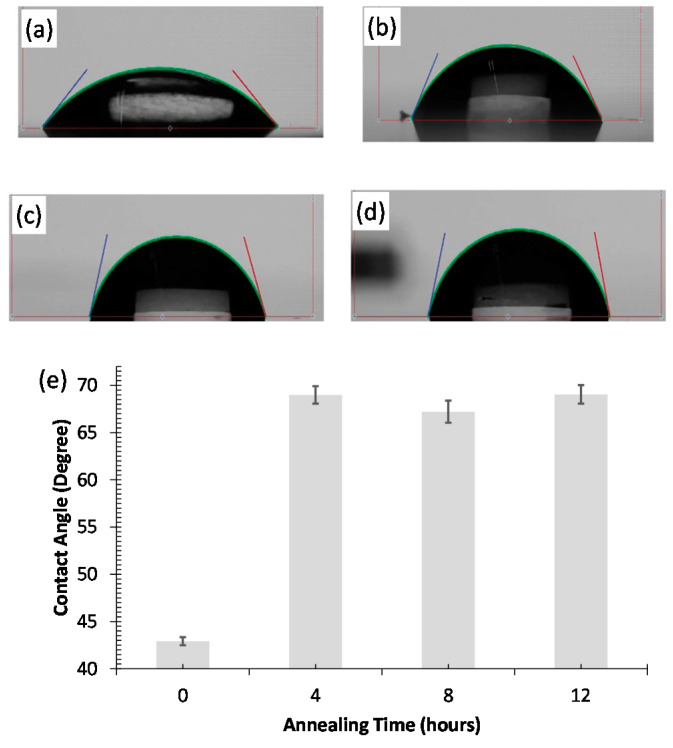
Images of water droplets on four COC wafers coated with GO (**a**) without baking, and (**b**–**d**) baked at a temperature of 100 °C for 4 h, 8 h, and 12 h, respectively. (**e**) The variation in the water contact angle on GO-coated COC wafers vs. the annealing time (four readings for each sample).

Due to the presence of oxygen functional groups that form hydrogen bonds, GO is known to have hydrophilic characteristics, resulting in low contact angles [22,23]. By reducing GO to rGO form, the hydrophobicity of the material is increased, hence increasing the contact angle between water droplets and GO surfaces [24]. By measuring the contact angle at various locations across the GO surface, this contact angle can be used to investigate the effect of baking time on the degree of reduction of the deposited GO. In this work, four fresh COC substrates are coated with GO after being treated with O_2_ plasma. Following that, three substrates are baked at a temperature of 100 °C for different durations (4 h, 8 h, and 12 h). Figure 2a–d show droplets of the same volume (5 µL) above the four samples. The four droplets have distinct contact angles. The effect of baking duration on the water contact angle is shown in Figure 2e. Without reduction, GO film has a contact angle of about 43°. After 4 h of annealing, the average contact angle is increased to around 68° and then almost saturates for 8 and 12 h annealing time. This experiment proves that heating the GO on COC substrate at 100 °C for a sufficient duration (i.e., ≥4 h) leads to the elimination of oxygen groups, making the material less hydrophilic, and thus achieving GO reduction.

#### 3.1.2. XRD and FESEM Analysis

**Figure 3:**
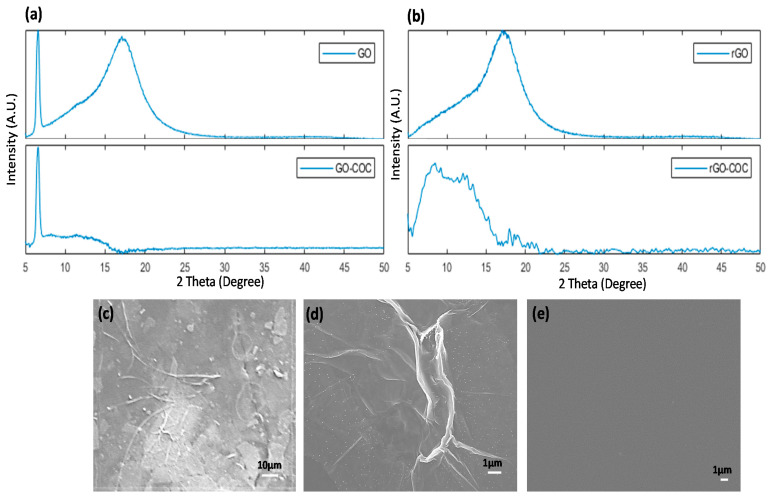
XRD pattern for (**a**) COC coated with pristine GO and diffractogram of the COC substrate subtracted from GO XRD, (**b**) GO reduced thermally for 12 h and diffractogram of the COC substrate subtracted from rGO XRD. (**c**–**e**) present microphotographs of the GO, rGO, and COC, respectively, captured using a field emission scanning electron microscope.

The results achieved in the previous section can be further verified by X-ray diffraction (XRD) analysis. Figure 3a,b shows the XRD pattern for two COC wafers coated with GO; one is not reduced, the other is annealed for 12 h at 100 °C, and both are compared to bare COC. The pristine GO shows a typical GO diffraction (Figure 3a) peak at 2θ = 7 with another intense peak at 2θ = 17 that is attributed to the COC wafer interacting with the X-ray beam [25]. Subtracting the COC diffractometer from that of GO leaves the typical GO peak at 2θ = 7. After reduction, the peak at 2θ = 7 drops in intensity and shifts to a greater 2θ. Huang et al. [26] demonstrated that the intense GO peak diminishes in intensity and shifts from 2θ = 10° to 25°, which coincides with the intense COC wafer peak. The XRD pattern for the 12 h of annealed GO (Figure 3b) shows no peaks at 2θ = 7°, the expected shift for rGO peak at 2θ = 25° is overshadowed by the COC peak. When subtracting the COC pattern from the rGO pattern, the resulting diffractometer reveals a broad low intensity peak at 2θ = 10, which is typical for thermally annealed rGO.

The deposited GO and rGO are further analyzed using Field Emission Scanning Electron Microscopy to determine their microstructures. Figure 3c–e show three SEM images of GO, rGO annealed for 12 h, and a bare COC substrate, respectively. The images in Figure 3c,d show a uniform distribution of the GO flakes with the presence of few wrinkles and defects and overlapping flakes. The SEM image of the bare COC substrate in Figure 3e reveals a uniform surface free of defects. The surface and microstructure features of GO and rGO are the results of the deposition process and thermal annealing.

#### 3.1.3. Raman Spectroscopy and AFM Analysis

**Figure 4:**
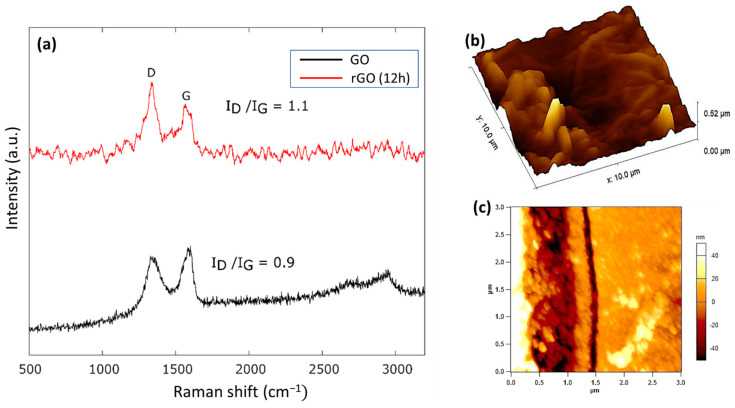
(**a**) Raman spectra of GO and rGO reduced for 12 h on COC substrate. (**b**) Surface morphology of GO layer as deposited on COC substrate; the root-mean-square roughness is measured at 62 nm. (**c**) AFM image of GO; the thickness of GO can be estimated as 100 nm.

The Raman spectra of a GO and rGO sample are revealed in Figure 4a. Raman spectroscopy analysis is conducted to investigate the structure of GO before and after thermal annealing. Two characteristic D (1340 cm^−1^) and G (1580 cm^−1^) peaks for GO are visible in Figure 4a. The presence of the D peak indicates disorders and defects in the GO film [27]. The ratio of the intensities of the D to G peaks is a measure of density of the disorders and defects in the form of sp^2^ to sp^3^ carbon ratio [28]. Before reduction, the D to G ratio is at 0.9, and after reduction the intensity ratio increases to 1.1. The increase in the intensity can be attributed to increased defects after the reduction process.

The GO layer is further analyzed using Atomic-forced microscopy (AFM), as shown in Figure 4b. The GO surface has a root-mean-square roughness of 62 nm. AFM is also used to measure the thickness of the deposited GO layer. As shown in Figure 4c, the thickness of GO can be estimated as 100 nm.

### 3.2. Electrical Characterization

#### 3.2.1. Switching Characteristics

**Figure 5:**
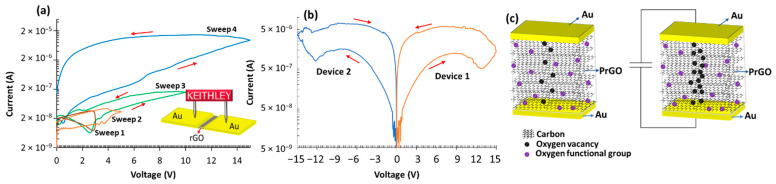
(**a**) *I-V* curves for 4 h rGO device generated by the parameter analyzer, multiple voltage sweeps are visible from 3 V to 15 V. (**b**) the *I-V* curve for two devices at ∓15 V sweeps, the hysteresis curve is mirrored, indicating unipolar switching. (**c**) A schematic of the switching mechanism takes place in the devices in (**a**,**b**), the GO layers are reduced by Joule heating, each subsequent sweep further reduces the GO flakes.

The MR devices fabricated in this work are characterized using a Keithley 4200-SCS parameter analyzer in sweeping mode to measure the *I-V* curves. Figure 5a presents the *I-V* characteristics of the fresh device reduced for 4 h. The test is carried out by applying consecutive voltage sweeps; the sweeps start from 0 V to Vmax, where Vmax is the maximum voltage specified for each sweep, ranging from 3 V to 15 V. As shown, at low voltage sweeps (3 V and 5 V) (Figure 5a), the MR has very high resistance (10^8^ Ohm) and shows minimal or no resistive switching behavior because of the very small current. Upon increasing Vmax to 10 V, the resistance of the device decreases with a very small OFF/ON ratio approaching 1. However, at 15 V sweep the device shows high resistance in the forward sweep and turns to the ON state with lower resistance during the reverse sweep, as in Figure 5a. Figure 5b shows the characteristics of two pristine devices reduced for 4 h, one under the application of −15 V and another under +15 V sweeps; the resulting *I-V* curve is the same for both devices with similar characteristics (i.e., current, resistance ratio). This switching behavior is unipolar switching since only voltage magnitude controls the resistance state, whereas in bipolar behavior the magnitude and polarity influence the resistance switching.

Understanding the resistive switching phenomena in GO-based MR devices and the mechanisms involved are essential [29]. Switching behavior describes the relation between the voltage magnitude and its polarity with resistive switching, while the switching mechanism describes the changes in the bulk GO flakes during resistive switching events. For GO-based MR devices, there are two main switching mechanisms in the literature [29]; metallic filamentary conduction is the most commonly used to explain resistive switching that takes place in GO devices [30,31]. This mechanism is dependent on the use of electrochemically active metal electrodes such as copper (Cu) in vertical and crossbar configurations [32]. When voltage is applied to the active electrode, the electrode material is dissolved, and the resulting ions migrate through the GO layer towards the passive electrode. Therefore, a conductive metallic filament is formed by the accumulating of ions, turning the MR ON. The filament is ruptured upon voltage polarity reversal returning the device to the OFF state [31,32]. MR devices that follow this mechanism typically exhibit abrupt and sudden current changes when the metallic filament is fully formed or ruptured [29]. The second mechanism is related to oxidation and reduction within the bulk GO layers. Graphene is a 2D material made of single-atom-thick flakes; graphene in this case is formed of electrically conductive sp^2^ bonds. GO, on the other hand, is formed by insulating sp^3^ bonds that are created by oxidizing the graphene flakes. When an electric field is applied, the oxygen functional groups in low conductivity areas are detached, converting the sp^3^ bonds to sp^2^ bonds, thereby locally increasing the conductivity of the GO film. This mechanism is more prevalent in planar devices and memristors using inert metal electrodes (i.e., gold and platinum) [33,34].

The device proposed in this work has a planar structure utilizing inert metal (gold) electrodes with a gap of 20 μm. Since the metallic filament conduction mechanism requires thin vertical devices (active layer thickness << 1 μm) [32,35] with electrochemically active electrodes (i.e., Al, Cu), the second mechanism–the oxidation-reduction in bulk GO–better fits the presented device, due to its relatively large electrode spacing and the use of inert gold electrodes. Moreover, as shown in Figure 5a,b, when resistance switching occurs, the current changes slowly and gradually from the high resistance state to the low resistance (OFF to ON) with no abrupt changes in current, confirming that oxidation-reduction reactions induced by the electric field [36] and Joule heating within the bulk GO film are responsible for resistive switching. Figure 5c presents a schematic that summarizes the reduction in bulk GO switching mechanism associated with the devices reported in Figure 5a,b.

#### 3.2.2. Tuning MR Switching Via Annealing Time

**Figure 6:**
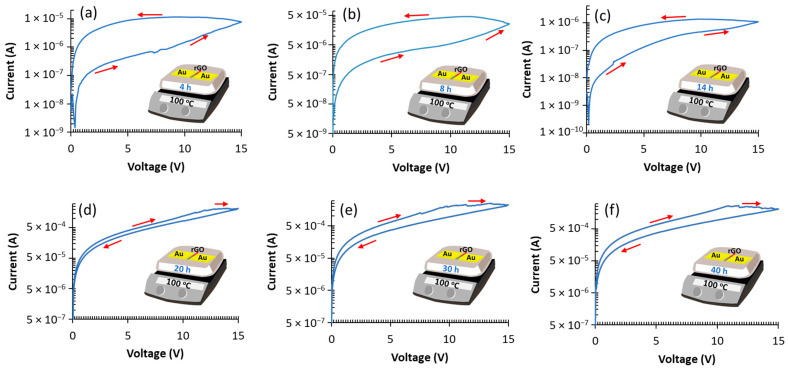
The switching characteristics for devices annealed over six different periods from 4 h to 40 h. (**a**–**c**) present switching from HRS to LRS, while (**d**–**f**) show switching from LRS to HRS.

In this work, tuning of the MR switching behavior is achieved by controlling the heating time of the fabricated devices, as presented in Figure 6. To elaborate, multiple fresh wafers are fabricated with different annealing times (4 h (a), 8 h (b),14 h (c), 20 h (d), 30 h (e), and 40 h (f)). All devices are tested using the same methodology by applying 15 V voltage sweeps. As shown in Figure 6, the fabricated devices have two different hysteresis curves; at a short reduction time (4 h, 8 h and 14 h), the memristor turns to a low resistance state. Around 20 h, a change in switching behavior occurs, and switching is reversed by transitioning from a low to a high resistance state for all reduction times greater than 20 h.

Thermal annealing of GO is a transient process. Here, we give insight into the impact of annealing time on the resistive switching behavior. Prolonged annealing time increases the conductivity of GO. The measured maximum resistance ratio varies with annealing time, as shown in Figure 6a–f. The maximum resistance ratio of 17 for 4-h samples is calculated at 7.5 V. As the annealing increases, the resistance ratio drops for 8-h samples, the maximum resistance ratio is 9.3 measured at an increased voltage of 8.5 V. At 14 h of annealing, the maximum resistance ratio drops to 2.9 at 9.8 V. Devices with 20-h reduction time have a maximum ON/OFF ratio of 1.6 measured at 12 V. At 30 h of annealing, a maximum ON/OFF ratio of 2.3 is realized at 9.8 V. Devices with 40-h reduction duration have a slightly higher resistance ratio of 2.4 when compared to 30 h, measured at 11 V. The decrease in the resistance ratio as annealing time increases (up to 14 h) can be attributed to the amount of oxygen functional groups within the graphene flakes. At a short annealing time (4 h), the graphene is still highly oxidized, which results in very high initial resistance, and as increasing voltage sweeps are applied, the GO is locally reduced by Joule heating produced by the applied voltage and the current passing through the device. At 8-h reduction, more oxygen groups are removed by thermal annealing during fabrication leaving fewer oxygen groups within the GO flakes to be removed by Joule heating induced by the electrical current during the writing process. Thus, the higher initial oxygen content allows for a higher resistance ratio. The decrease in the resistance ratio for 14 h devices in Figure 6c is also linked to the amount of oxygen functional groups present within the rGO flakes. After 14 h of annealing, more oxygen groups are removed. Since the ON/OFF ratio is created by the local reduction of GO by Joule heating, the lack of oxygen groups within the GO flakes allows for limited resistive switching.

As presented in Figure 6d–f, at longer reduction periods (20 h and more) the MR resistive switching behavior is reversed, the device starts at low resistance, and when a threshold voltage is reached during a voltage sweep, the device switches to a high resistance state. The threshold voltage is tied to the degree of reduction of the GO flakes; at 20 h of annealing, resistive switching starts at 13 V, and drops to 10 V for the 30- and 40-h samples. Moreover, the devices reduced for 20 h or more show significantly higher current levels in both the forward and backward sweeps. At 14 h reduction time and less, the maximum current reached is below 1 μA, however, Figure 6f shows current levels that are about 100 times higher at a current peak of 2.7 mA for the 40 h samples. The resistance ratio is also lower; at 20 h, the maximum average resistance ratio occurs is 1.6 which occurs at 12 V. As annealing time is increased to 30 h, the threshold voltage drops to 10 V and the OFF/ON ratio increases to 2.3 at 9.8 V. At 40 h of reduction, the *I-V* curve shown is similar to that of 30 h with minuscule changes; the similarities are due to reduction process of graphene oxide. Thermal annealing of GO at low temperatures (100 °C) is a slow process, and therefore the 30 h and 40 h devices produce comparable results. Compared to the mechanism explained in Section 3.2.1 for devices reduced for ≤14 h, a different mechanism occurs for 20 h reduction or more. To elaborate, in such devices, the GO is highly reduced, but some oxygen groups still exist. By applying a high voltage sweep (i.e., 15 V), the remaining oxygen species within the bulk GO form a resistive barrier by local oxidation of GO, thus increasing the resistance. The GO is no longer being reduced by voltage as in the case of 4–14 h, but the diffusion of remaining oxygen groups is responsible for resistive switching [3].

It is worth mentioning that reduction of GO at 100 °C occurs at slow rate, which enables the tunable resistive switching by varying the reduction time, which is the main novelty of this work. Higher annealing temperatures accelerate the reduction process. Nonetheless, if the GO is reduced too rapidly at elevated temperatures, this may alter the switching behavior such that the behavior observed in this study may no longer be observed. Thus, annealing at higher temperatures reduces the ability to control the reduction process and achieve precise device resistance tuning. As evident in the work of Slobodian et al. [37], where they measured the reduction of GO versus annealing temperature in terms of weight loss percentage, the reported results show the rate of reduction changes significantly with temperature especially above 100 °C. At 100 °C, it is revealed that the rate of reduction is enough to observe the effects via fine reduction of the GO film.

#### 3.2.3. Tuning MR Switching via DC and Sweep Voltage

**Figure 7:**
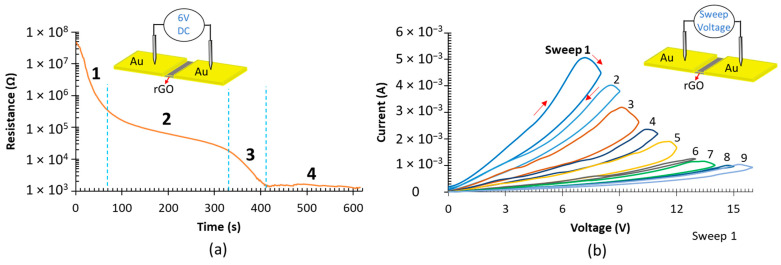
Tunable MR switching behavior. (**a**) Fresh device annealed for 12 h is tested under the application of 6 V DC bias. (**b**) *I-V* curves for device in (**a**) after the application of the DC voltage bias.

To further investigate the switching characteristics of the GO-based MR devices fabricated in this work, constant DC voltage bias is applied to a fresh device, and the resistance response is logged over time. Figure 7a presents the resistance over time, under the application of 6 V DC voltages, for a pristine device annealed at 100 °C for 12 h. As shown, in 400 s, the device resistance drops from 47 MΩ to 1.3 KΩ and then reaches a saturation resistance state. The steep decline in the resistance of the active rGO layer within the memristor indicates localized reduction by the current-induced Joule heating effect. The final stagnation resistance of the device electrically reduced by joule heating in Figure 7a resembles the devices thermally reduced for 20 h and more. To elaborate, the same device is tested with linear voltage sweeps and the resultant *I-V* curves are plotted in Figure 7b. It is clear that the switching direction of the device is reversed to be from low resistance state to high resistance state in an analog manner. As depicted, under the application of a voltage sweep that is equal to or exceeds the switching threshold voltage, the resistance increases by Joule heating, inducing solid phase dissolution of oxygen functional groups [33]. By increasing the resistance, the current drops and a high voltage level is needed to recreate the Joule heating effect. By increasing the write voltage, a new high resistance state can be written. This novel tunable behavior of the MR device is considered a great asset as it broadens the application horizon of the device.

**Figure 8:**
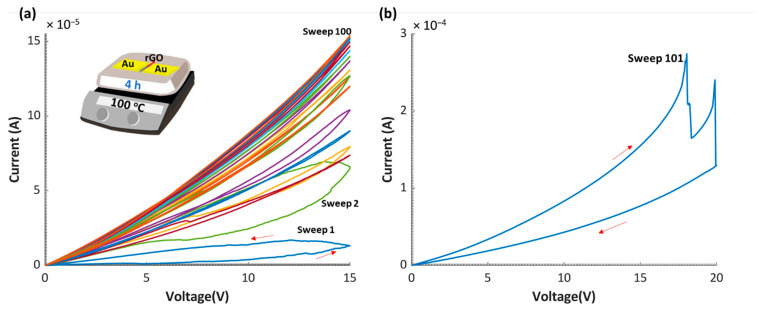
Tunable MR switching behavior. (**a**) A fresh device annealed for 4 h is tested for 100 cycles under the application of 15 V sweep. (**b**) *I-V* curve under the application of 20 V sweep for the same device after performing the test in (**a**).

The electrical behavior of the memristor devices reported in this work is further investigated by applying 100 consecutive voltage sweeps at 15 V, across a 4 h reduced device. As shown in Figure 8a, during the first sweeps, the memristor exhibits resistive switching behavior, where the resistance decreases between the forward and the backward sweeps. After each sweep, the resistance decreases when compared to the previous sweep. Additionally, the ON/OFF ratio decreases until the sixth sweep, where the resistances in the forward and backward sweeps are equal (ON/OFF ratio = 1). From the 6th sweep until the 14th sweep, the resistance decreases further with the same resistance ratio (ON/OFF ratio = 1). After the 14th sweep, the device no longer behaves as a memristor, and all consequent sweeps coincide. After that, as presented in Figure 8b, a 20 V dual sweep is applied to the same device; the resistance increases at 18 V, and the device switches to a higher resistance state. Therefore, it can be concluded that after around 14 sweeps at 15 V, a 4 h device will change switching behavior and the resistance can increase with future sweeps via applying higher voltage magnitude.

## 4. Conclusions

In conclusion, this work presented a novel thermally reduced graphene oxide memristor in a planar Au/rGO/Au configuration. The presented device had a unique tunable resistive switching behavior controlled by adjusting the reduction time of the GO layer. At shorter reduction times, the device switched from OFF to ON, and increasing the reduction time reversed the switching to be from the ON to OFF state. In both stages, the devices exhibited analog switching behavior. The use of symmetric gold electrodes resulted in a unipolar switching behavior where the voltage amplitude controlled the switching and writing process irrespective of polarity. The resistive switching phenomenon was linked to the oxidation-reduction of the oxygen species within the bulk GO flakes. Moreover, a unique switching behavior was achieved by further reducing the MR devices using a suitable DC bias. This led to reversing the switching direction of the MR resistance state and achieving multi-state switching characteristics. The novel methodologies reported in this work provided new insights to develop rGO-based MR devices with highly tunable characteristics to adapt the target applications.

## Data Availability

All data generated or analyzed during this study are included in this published article.
